# Perceptions of an older patient on the role of the family doctor in health promotion: a qualitative case study

**DOI:** 10.1186/1752-1947-7-57

**Published:** 2013-02-28

**Authors:** Ludmila Marcinowicz, Teresa Pawlikowska, Adam Windak, Slawomir Chlabicz

**Affiliations:** 1 Department of Family Medicine and Community Nursing, Medical University of Bialystok, Mieszka I 4 B, Bialystok 15 054, Poland; 2 Warwick Medical School, The University of Warwick, Coventry, UK; 3 Department of Family Medicine, Jagiellonian University Medical College, Krakow, Poland

**Keywords:** Case studies, Health promotion, Older people, Primary health care

## Abstract

**Introduction:**

Health promotion and disease prevention are important aspects of primary health care. However, limited data are available concerning the opinions of older patients towards the respective services offered by family doctors. The aim of the present study was to evaluate an older patient's perception of the role of the family doctor in promoting his health, and identify those components that are difficult to examine in quantitative research.

**Methods:**

A qualitative case study of an 80-year-old man using an in-depth interview was carried out. The interview transcript was analyzed thematically. Our patient was an 80-year-old university-educated man, with stable social and financial circumstances, living with his wife. He had retired early on grounds of ill health (tuberculosis) and had received a disability pension prior to formal retirement. At the time of the interview, his medical problems included mild prostatic hypertrophy, scoliosis and hypertension. He considered his health status to be satisfactory. He had changed family doctor five years prior to the interview, as he had been dissatisfied with the care provided.

**Results:**

We found that our patient expected the family doctor to be aware of, and to discuss, the following issues: physical activity, diet, management of stress and mental health, use of alcohol and tobacco, personal hygiene, health screening, use of medication, and social activity. At the same time, our patient perceived the doctor's role as supplementary to his own in terms of the appraisal and maintenance of his health.

**Conclusions:**

Our findings provide evidence of what is important in the promotion of health among older people.

## Introduction

Health promotion and disease prevention are key aspects of primary care. Patients expect their family doctor to help prevent or delay disease as well as to treat existing disorders [1-3]. Family doctors themselves consider health promotion and disease prevention to be important aspects of their work [4,5]. However, they report numerous obstacles to their effective participation in this field of activity, such as lack of time and insufficient financial reimbursement [6]. There are growing fears in the profession that preventive measures may not result in the anticipated benefits. There is also evidence of the effectiveness of strategies for promoting health in old age [7]. However, research has identified a tendency to treat older people as a homogenous group without considering age, education, and social and functional status [8]. Interventions may be considered worthless if they are inconsistent with the beliefs, attitudes and expectations of older patients. Evidence-based guidelines on health promotion for older people recommend giving older people a voice and involving them in projects [9]. This approach is consistent with the modern view of the patient as an evaluator of care and a potential ‘agent of change’ [10].

The aim of the present qualitative case study was therefore to understand and evaluate an older patient’s perception of the role of the family doctor in health promotion, and identify those components that are difficult to detect in quantitative research.

## Methods

A qualitative case study was conducted according to the methodology described by Stake [11,12] and Patton [13]. According to Stake [12] ‘[A] case study is not a methodological choice but a choice of what is to be studied’.

The present case study was conducted within the context of a qualitative study designed to explore the experience of general practice of patients aged 65 years and above. The study was conducted in two demographically diverse Polish cities (Bialystok (population 295,000) and Krakow (population 755,000)). Purposive sampling was used to maximize variation in the data (age, gender, education and health problem). Recruitment was halted after 30 interviews when data saturation was reached. The sample consisted of 18 women and 12 men, aged 65 to 87 years. Seven participants had a basic education, 10 had a vocational education, seven had received secondary education and six had received higher education. The majority (21) were married.

A trained qualitative researcher (LM, who is not a family doctor) carried out individual in-depth interviews at patients’ homes, during which they were encouraged to talk freely about the care provided by their family doctor [14]. Interviews were based on an interview guide (see Appendix 1), lasted approximately one hour and were recorded on tape and then transcribed.

During the interview process the respondent featured in this report was identified as a ‘typical case’ [13]. The representativeness of this case was established on the basis of the scope and typicality of the problems discussed during the interview and the expectations expressed. According to Patton ‘the purpose of a qualitative profile of one or more typical cases is to describe and illustrate what is typical to those unfamiliar with the setting - not to make generalized statements about the experiences of all participants. The sample is illustrative not definitive’ [13]. This interview therefore became the subject of the present separate in-depth analysis. The special importance of this case was associated with the fact that it was information rich [13].

The interview transcript was subjected to a thematic analysis. First, themes referring to health promotion were noted and grouped into different categories independently by two authors from different professional backgrounds: a researcher and a family doctor. Next, the major themes were analyzed and compared according to existing themes on health promotion. Additionally member checking was used as described by Stake [11]. The initial report was therefore presented to our respondent, who confirmed the interpretation of the data as being correct.

The study was approved by the Ethics Committee of the Medical University of Bialystok, Poland (approval number: R-I-002/251/2009).

## Case presentation

Our patient was an 80-year-old university-educated man, with stable social and financial circumstances, living with his wife. He had retired early on the grounds of ill health (tuberculosis) and had received a disability pension prior to formal retirement. At the time of the interview, his medical problems included mild prostatic hypertrophy, scoliosis and hypertension. He considered his health status to be satisfactory. He had changed family doctor five years prior to the interview, as he had been dissatisfied with the care provided.

## Results

### Patient’s perspective

Our patient’s opinions concentrated on the need for the family doctor to promote several specific health behaviors, including physical activity, diet, stress and mental health, alcohol and tobacco, personal hygiene, health screening, prescription of medication and social activity.

### Physical activity

Our patient expressed his perception of the role of the family doctor in this area as follows:

‘Physicians should emphasize the need to apply preventive measures and behaviors in everyday life (…). You need a bit of movement, and this is what the physician should expose, ask “Do you go for a walk?”’

### Diet

Our patient emphasized that he tried to maintain a healthy diet, and drew attention to the role of the family doctor in this area:

‘They [doctors] should ask: What do you eat? Do you eat fatty foods? What meat do you eat? It seems very important to me.’

### Stress and mental health

Our patient also expressed the view that the family doctor should advise patients on how to cope with stress, and that the doctor should recognize their emotional state:

‘There is stress. Whether a man wants it or not, he has to think about it. The doctor should give some examples that another patient with the disease was cured or the disease was stopped, not fictitious but from his own medical practice. I think this is important. Emphasizing the optimistic prognosis - cure or disease control. (…) The doctor should enter into the role of the psychologist, feel it somehow.’

### Alcohol and tobacco

Our patient also referred to alcohol misuse and tobacco smoking among older patients. He was convinced that the family doctor had more power in persuading patients to stop smoking than the mass media:

‘The doctor should emphasize - this is such a truism - the importance of excluding alcohol and cigarettes. Doctors should ask: Do you smoke? The effect of hearing it from the doctor is greater than from an advert which says: Let’s stop smoking.’

### Personal hygiene

Our patient also expressed the opinion that the family doctor should educate patients concerning the issue of personal hygiene.

‘Hygiene. Of course, this is a very personal matter, but if the doctor notices poor hygiene while examining the patient, then he should mention it. (…) Yes, the doctor should say something. Gently, so as not to offend anyone, as this apparently obvious matter could generate conflict.’

### Health screening

Our patient also expected the family doctor to refer him for periodic screening investigations.

‘There should be a kind of a doctor’s schedule, dates to have tests, that is blood and urine tests and an examination of the heart.’

### Prescription of medication

Our patient referred to the overprescription of medication to older patients:

‘I can understand that pharmacology and medication are important, but I have come to the conclusion that too many tablets are prescribed to the elderly. They don’t even take them all, or use them, but at the pharmacy you see a woman carrying a bag full of these tablets. The doctor should be very moderate about prescribing.’

### Social activity

Despite describing himself as being open and willing to communicate with others, our patient expressed the need for contact with his family doctor:

‘I am, as they say, a social creature in the sense that I like being in touch with people, talking to people from various professions, and I have diverse interests. (…) It seems to me that there must be some link between the doctor and myself that we can talk about, even something small like the fact that we live in the same district.’

The respondent also referred to the ethos of family doctors:

‘It seems to me that the very fact that the term family doctor exists is good. This creates such warmth - not literally familial - but such a warm atmosphere. Family - the word itself is binding. The doctor is the most important person to a man, not his employer, even though he pays his wages, but the doctor; it is not a cliché to say that health is the most important thing for a man.’

At the same time, our patient perceived the doctor’s role as supplementary to his own in terms of the evaluation, control, and maintenance of his health: ‘…because the family doctor is somehow a continuation of the doctor I myself am’.

## Discussion

The choice of using a case study as a research technique may be controversial in terms of the hierarchy of evidence. It is not clear to what extent a single case can provide more general information, however according to Stake [11] each case is both individual and general, since we can usually find both specific and typical elements. The principal epistemological value of a case study is well expressed by the Latin maxim ‘non multa sed multum’ (not many but much).

Qualitative case studies are uncommon in medical research. However, novel research issues necessitate a flexible approach to the selection of methods and techniques. A common rationale for the performance of a case study is that an in-depth analysis of a single case will contribute to the understanding of a wider issue. Therefore, the choice of case research makes an essential contribution to increasing our understanding [12]. In the present study, assessment of an individual case provided a more comprehensive picture of older patients’ perceptions of the role of the family doctor in health promotion. The language used by our patient to describe his expectations provides specific information concerning the impact of health promotion interventions on older patients. By getting to know not only a patient’s opinions, but also his feelings, experiences, behaviors and expectations, we gain both understanding and practical benefits. Firstly, it shows the need for an individual approach to patients and identifying their expectations of care. Secondly, it provides practical information on how to deal with this during the visit. Lastly it emphasizes the need for a flexible mindset; health promotion is relevant even at 80 years old!

Member checking was used to substantiate the quality of the data analysis: our patient confirmed the accuracy of the interpretation of his opinions and expectations. This did not negate the principle that the positions and roles of the respondent and the researcher are inherently different [15].

The present study provides new insights by assessing the issue of health promotion from the perspective of a patient. Our patient felt that the family doctor has a role in the maintenance of the physical and mental skills of older patients and in encouraging them to form social bonds within their communities. He expressed the opinion that the family doctor should be an educator concerning the issues of smoking, alcohol use, physical activity, nutrition, medication and coping with stress. He also referred to the role of the family doctor regarding the issue of personal hygiene, which is rarely discussed in the literature. Our patient himself touched upon key health promotion issues described in a variety of studies [1-3,8]. This case study highlights the importance of a flexible approach to consulting; our patient himself articulates his expectation of health promotion at age 80.

Health promotion is a very broad term and takes into account a wide range of factors that encompass the health of an individual. This paper concerns only individual lifestyle factors and the health services of a family doctor. Our patient perceived the doctor as a ‘key person’ in health promotion. His expectations are consistent with evidence-based guidelines on health promotion for older people. An important recommendation is to ‘enable older people to improve their independence and autonomy through increasing practical know-how’ [9].

Our patient in the present study was relatively well from the perspective of his physical and mental condition and he had stable family, social and financial circumstances. He took personal responsibility for his health, was conscious of negative health behaviors and was willing to cooperate with his family doctor.

## Conclusions

A case study can provide a wider perspective of different problems concerning health care. This study shows that the promotion of health is seen as important by older people themselves. A family doctor is expected to recognize health needs through active discussion, to offer advice concerning lifestyle, enhance positive health behaviors, and provide support for patients.

## Consent

Written informed consent was obtained from the patient for publication of this case report and any accompanying images. A copy of the written consent is available for review by the Editor-in-Chief of this journal.

## Appendix 1

Open-ended interview questions included:

What is most important for you in the overall care given by your family doctor?

What do you mean when you say you are satisfied with a visit to your family doctor?

What do you like most in the family doctor behavior?

Which of your family doctor’s behaviors did you dislike?

## Competing interests

The authors declare that they have no competing interests.

## Authors’ contributions

LM was involved in the original idea, design, analysis and interpretation of the data, and wrote the case report. TP contributed to interpretation and critical revision for important intellectual content and final editing of the manuscript. AW and SC interpreted the data and were involved in drafting the manuscript. All authors read and approved the final manuscript.
